# Antiphospholipid Antibody and Risk of Retinal Vein Occlusion: A Systematic Review and Meta-Analysis

**DOI:** 10.1371/journal.pone.0122814

**Published:** 2015-04-28

**Authors:** Wei Zhu, Yan Wu, Ming Xu, Jin-Yu Wang, Yi-Fang Meng, Zheng Gu, Jiong Lu

**Affiliations:** 1 Department of Ophthalmology, Changshu N_O_.2 People's Hospital, Changshu, China; 2 Department of Ophthalmology, The First People's Hospital of Kunshan Affiliated with Jiangsu University, Suzhou, China; Massachusetts Eye & Ear Infirmary, Harvard Medical School, UNITED STATES

## Abstract

**Background:**

Retinal vein occlusion (RVO) is a common retinal vascular disease and it is one of the most frequently reported causes of visual damage and blindness in the elderly. The current study investigated the potential association between antiphospholipid antibodies (APLA) and RVO risk by conducting a meta-analysis of case–control studies.

**Methods:**

A systematic literature search of Pubmed and Embase databases was conducted in August 1^st^, 2014. Odds ratios (ORs) were used to evaluate the associations between APLA and the incidence of RVO. A random-effects model was obtained for the quantitative synthesis.

**Results:**

A total of 11 studies were included in this meta-analysis. A meta-analysis of all studies assessing the risk of RVO revealed that APLA was associated with a statistically increased risk of RVO incidence (OR = 5.18, 95% CI = [3.37, 7.95]). The association between anticardiolipin antibodies (ACA) and the risk of RVO was significant (n =8, OR = 4.59, 95% CI = [2.75, 7.66]). However, the association between lupus anticoagulants (LA) and risk of RVO was non-significant (n = 5, OR = 3.90, 95% CI = [0.99, 15.37]). No significant publication bias was found in the 11 selected studies.

**Conclusion:**

APLA was significantly associated with the risk of RVO. Advanced analyses showed that ACA rather than LA affected the risk of RVO. Additional well-designed and well-conducted epidemiological studies are required to further our understanding of the relationship between APLA and RVO risk.

## Introduction

Retinal vein occlusion (RVO) is a common retinal vascular disease, and it is one of the most frequently reported causes of visual damage and blindness in the elderly [1]. Central retinal vein occlusion (CRVO) and branch retinal vein occlusion (BRVO) are the most common subtypes of RVO. CRVO and BRVO reduce an individual’s functioning and quality of life, especially if macular edema appears [2]. Different therapies, including intravitreal injection of anti-VEGF agents, are applied, but the prognosis of visual acuity remains poor [3].

In general, RVO is a multifactorial disease, and the causes of RVO are quite complex [4]. Cardiovascular and hematological abnormalities, such as hypertension, arteriosclerosis, high blood viscosity and hemodynamic abnormalities, are associated with RVO [5,6]. Additionally, trauma and oral contraceptives are associated with an increased risk of RVO [7,8]. RVO is a multifactorial disease in which the abnormalities in vascular factors and hemodynamic components are important etiological factors.

Antiphospholipid antibodies (APLA) include two main types, lupus anticoagulants (LA) and anticardiolipin antibodies (ACA). APLAs are acquired autoantibodies against phospholipid–protein complexes, which act as important autoantibodies in anti-phospholipid syndrome. Previous studies indicated that APLA is associated with pregnancy [9], thrombosis [10] and stroke [11]. Several studies also reported a potential association between the APLA and risk of RVO [12–22], but no accordant conclusion was obtained. The current study investigated the potential association between APLA (both ACA and LA) and RVO (both CRVO and BRVO) risk using a meta-analysis of case–control studies.

## Methods

This systematic review and meta-analysis was conducted following the guidelines of Meta-analysis of Observational Studies in Epidemiology [23], and it is reported according to the Preferred Reporting Items for Systematic Reviews and Meta-Analyses (PRIAMA) guidelines [24].

### Search strategy

A systematic literature search of Pubmed and Embase databaseswas conducted independently in August 1^st^, 2014 by two reviewers (WZ and YW) for all relevant studies on the association between APLA and RVO risk. The medical subject heading (MeSH) terms and key words used in the search included “retinal vein occlusion”, “retinal vein obstruction” combined with “antiphospholipid antibody”, “lupus anticoagulants” and “anticardiolipin antibody”. The title and abstract of studies identified in the search were reviewed by two authors independently (WZ and YW) to exclude studies that were not associated with the main outcome of this study. The full text of the remaining articles was examined for the final inclusion of the relevant studies. We also manually searched the reference lists for possible valuable studies. When incomplete information was available, attempts were made to contact the corresponding authors of the studies for additional information by writing an e-mail to the corresponding author.

### Inclusion and Exclusion Criteria

We set the following criteria to identify the adequate studies included in this meta-analysis:

1. A case-control study design was obtained for the relevant studies.
2. The association between APLA (ACA, LA or combined) and RVO (CRVO, BRVO or combined) risk was reported.

The following major reasons were used for the exclusion of studies:

1. No outcomes of interests were reported.
2. Absence of raw data in a useful format.

### Data Extraction

Data in each included study was extracted by two independent reviewers (WZ and YW) using the same standardized method. Any disagreements were settled by additional reviewers until a **consonance** was reached. Information obtained from each study included the first author, year of publication, types of APLA and RVO, and the numbers and characteristics of the participants in each study. The incidence data in different groups were extracted for the following meta-analysis.

### Statistical analysis

We used the random-effects model for quantitative synthesis because all of the included studies were observational in nature. Odds ratios (ORs) were used to evaluate associations between APLA and risk of RVO. The heterogeneity between studies was estimated using *χ* ^*2*^ and *I* ^*2*^ tests. A P value less than 0.10 or an *I* ^*2*^ over 50% was considered suggestive of significant heterogeneity because tests for heterogeneity lack power. Once heterogeneity was noted in this study, the sources of heterogeneity were investigated using subgroup analyses by stratifying original estimates according to the study characteristics (as described above). Publication bias was qualitatively assessed using visual inspection of funnel plots of the logarithmic OR vs. their standard errors and quantitatively assessed using Begg and Egger tests [25,26]. Analyses were performed in Stata Version 8 (StataCorp Stata Statistical Software: release 12.0, Stata Corporation, College Station, TX).

## Results

### Literature search

**Fig 1:**
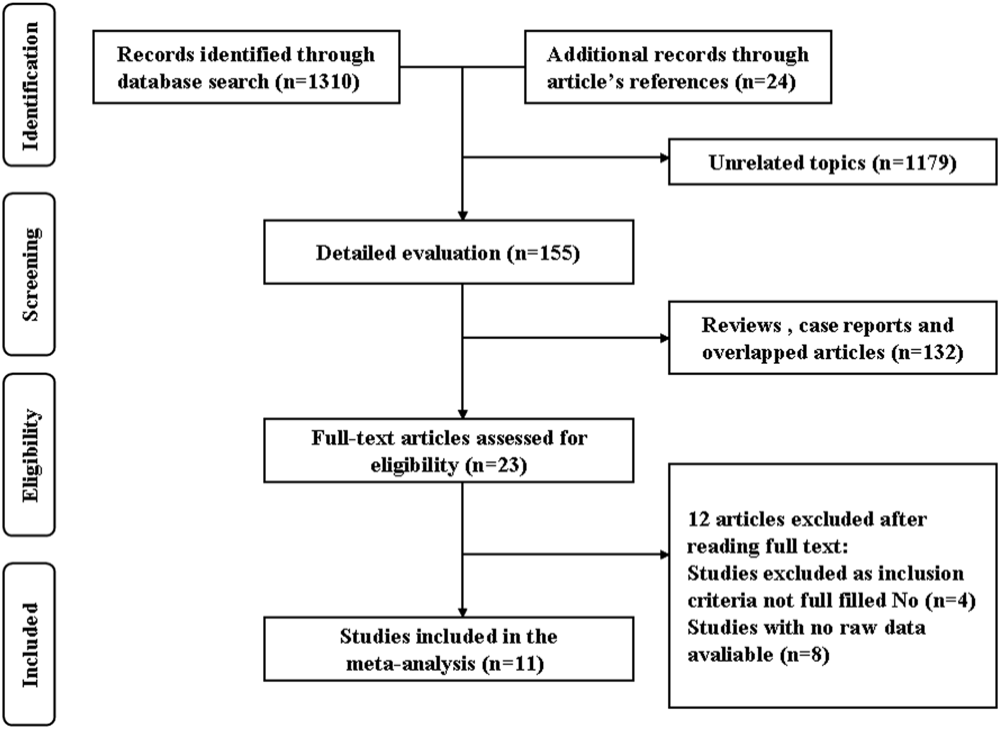
PRISMA diagram of included studies.

The Pubmed search yielded 659 potentially relevant articles, and the EMBASE search yielded 641 articles. Twenty-four additional records were identified through a review of article references. A total of 1179 records of these articles were excluded based on the title and abstract review because they were clearly irrelevant to the objectives of this meta-analysis. In the detailed evaluation, after excluding 132 reviews, case reports and overlapped articles, 23 full-texts were obtained for full-text review. Finally, 11 studies were included in this meta-analysis [12–22] after 12 articles were excluded (4 studies were excluded because inclusion criteria not fulfilled, and 8 studies without available raw data were excluded). No unpublished data were searched and included in this study. The flow diagram is presented in Fig 1.

### Characteristics of included studies

The characteristics of these studies are shown in Table 1. The earliest study was published in 1994 and the latest study in 2014. Four studies were performed in the Europe, four studies were performed in America, three studies were performed in Asian populations, and one study was performed in Africa. When a control group source was considered, both healthy controls and the individuals without RVO were applied. Both IgG and IgM antibodies were analyzed in most included studies.

**Table 1:** Characteristics of included studies assessing APLA and the risk of RVO.

Author	Publication year	Country	All subjects	Control source	Age (year)	Antibody type	Diagnostic methods
Maaroufi RM et al	2004	Tunisia.	113	Healthy control	11–63	IgG, IgM	Fundus fluorescein
Risse F et al	2014	Germany	179	Healthy control	62.9	NA	Clinical History
Cobo-Soriano R et al	1999	Spain	80	No RVO	55	IgG, IgM	Recently diagnosed
Adamczuk YP	2002	Argentina	141	Healthy control	49	IgG, IgM	Clinical History
Van Cott E	2004	USA	60	No RVO	57	IgG, IgM	Clinical History
Atchaneeyasakul LO	2005	Thailand	131	Healthy control	54	IgG	Clinical History
Abu el-Asrar AM	1996	Saudi Arabia	87	Healthy control	38.7	IgG, IgM	Fundus fluorescein
Glacet-Bernard A	1994	France	106	No RVO	47	IgG, IgM	Clinical History
Glueck CJ	2012	USA	237	Healthy control	55	IgG, IgM	Clinical History
Abu El-Asrar AM	1998	Saudi Arabia	131	Healthy control	51	IgG, IgM	Recently diagnosed
Marcucci R	2001	Italy	200	Healthy control	57.5	IgG, IgM	Clinical History

### Risk of RVO

**Fig 2:**
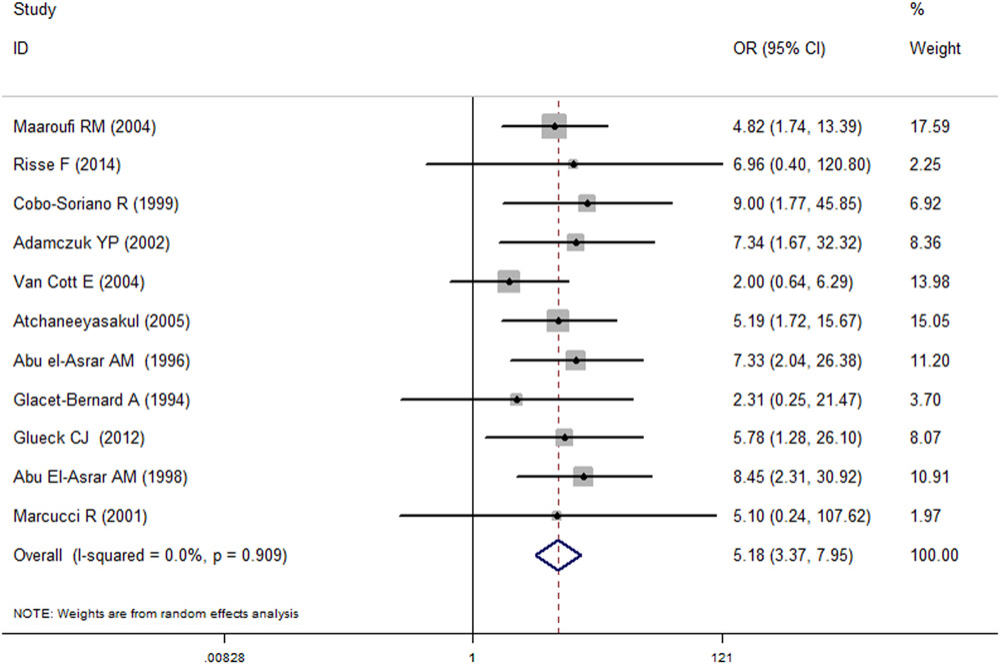
The forest for detection of APLA as a risk factor for RVO.

Meta-analysis of all studies assessing the risk of RVO revealed that APLA was associated with a statistically significant increase in RVO incidence (OR = 5.18, 95% CI = [3.37, 7.95]). (Fig 2). There was no considerable heterogeneity observed across studies (I^2^ = 0.00%, P = 0.909). Advanced subgroup analyses by locations showed that APLA was associated with an increased risk of RVO in Europe (n = 4, OR = 5.72, 95% CI = [1.88, 17.40], *I* ^*2 =*^ 0.00, P = 0.812), America (n = 3, OR = 3.87, 95% CI = [1.68, 8.88], *I* ^*2*^ = 0.12, P = 0.322), Asia (n = 3, OR = 6.65, 95% CI = [3.29, 13.42], *I* ^*2*^ = 0.00, P = 0.840) and Africa (n = 1, OR = 4.82, 95% CI = [1.74, 13.39]). When the control group source was considered, significant associations between APLA and increased risk of RVO were detected in both healthy controls (n = 8, OR = 6.11, 95% CI = [3.73, 10.01], *I* ^*2*^ = 0.00, P = 0.998) and individuals without RVO (n = 3, OR = 3.21, 95% CI = [1.24, 8.29], I^2^ = 12.70, P = 0.318). Furthermore, these results were significant in the studies with over 100 subjects (n = 8, OR = 5.62, 95% CI = [3.34, 9.46], *I* ^*2*^ = 0.00, P = 0.990) and less than 100 subjects (n = 3, OR = 4.61 CI = [1.75, 12.13]). No significant heterogeneity was detected in any of the subgroup analyses (Table 2).

**Table 2:** Subgroup analysis assessing APLA and the risk of RVO by characteristics of included studies.

Subgroup	No. of studies	Summary OR (95% CI)	Heterogeneity	
			*I* ^*2*^ score (%)	P value
All studies	11	5.18 [3.37, 7.95]	0.00	0.909
Geographic location				
Europe	4	5.72[1.88, 17.40]	0.00	0.812
America	3	3.87 [1.68, 8.88]	0.12	0.322
Asia	3	6.65 [3.29, 13.42]	0.00	0.840
Africa	1	4.82 [1.74, 13.39]	N/A	N/A
Control source				
Healthy controls	8	6.11[3.73, 10.01]	0.00	0.998
Without RVO	3	3.21 [1.24, 8.29]	12.70	0.318
No. of subjects				
> 100	8	5.62 [3.34, 9.46]	0.00	0.990
< 100	3	4.61 [1.75, 12.13]	0.00	0.909

**Fig 3:**
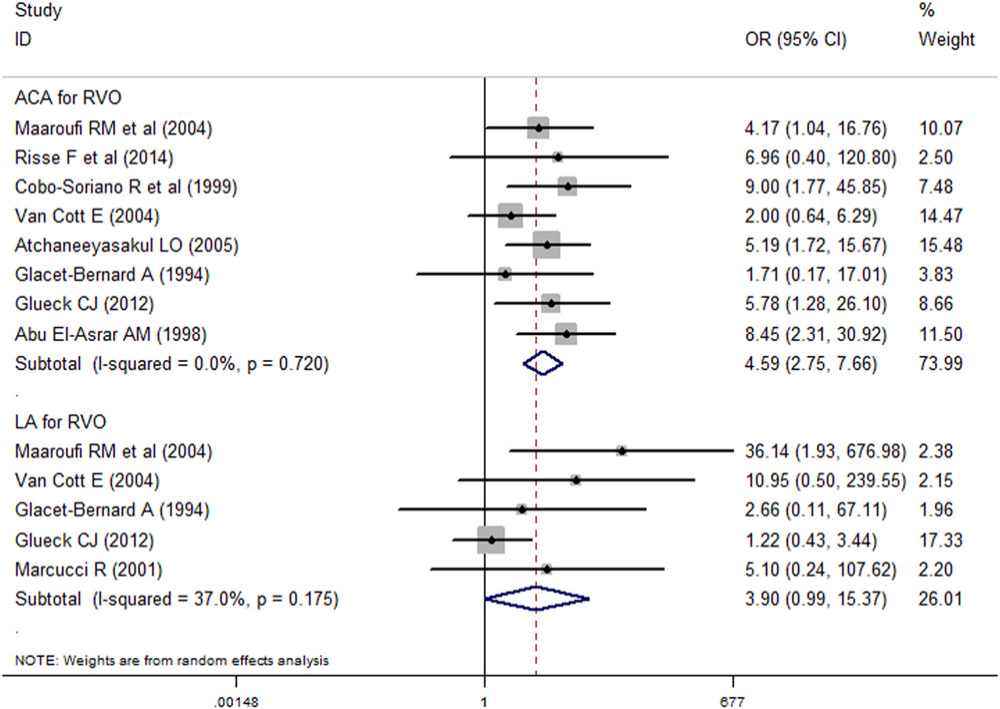
The forest for detection of ACA and LA as risk factors for RVO.

We conducted subgroup analyses by APLA and RVO subtypes (Fig 3). The association between ACA and RVO risk was significant (n = 8, OR = 4.59, 95% CI = [2.75, 7.66], *I* ^*2*^ = 0.00, P = 0.720). However, the association between LA and RVO risk was not significant (n = 5, OR = 3.90, 95% CI = [0.99, 15.37], *I* ^*2*^ = 37.00, P = 0.890). In general, the associations of APLA and both CRVO (n = 7, OR = 4.40, 95% CI = [2.06, 9.36], *I* ^*2*^ = 0.00, P = 0.644) and BRVO (n = 4, OR = 10.09, 95% CI = [3.99, 25.50], *I* ^*2*^ = 0.00, P = 0.413) were significant. When the associations of ACA and the two subtypes of RVO were considered, both ACA and CRVO risk (n = 8, OR = 4.59, 95% CI = [2.75, 7.66], *I* ^*2*^ = 0.00, P = 0.854) and ACA and BRVO (n = 4, OR = 7.50, 95% CI = [1.61, 35.03], *I* ^*2*^ = 37.00, P = 0.890) were significant. However, no significant association between LA and CRVO was detected (n = 4, OR = 1.75, 95% CI = [0.69, 4.21], *I* ^*2*^ = 0.00, P = 0.586). No correlation between LA and BRVO risk was found. Similarly, no significant heterogeneity was detected in any of the subgroup analyses in the above comparisons (Table 3).

**Table 3:** Subgroup analysis assessing APLA and the risk of RVO by subtypes of APLA and RVO.

Subgroup	No. of studies	Summary OR (95% CI)	Heterogeneity	
			*I* ^*2*^ score (%)	P value
APLA for RVO	11	5.18 [3.37, 7.95]	0.00	0.909
APLA for CRVO	7	4.40 [2.06, 9.36]	0.00	0.644
APLA for BRVO	4	10.09 [3.99, 25.50]	0.00	0.413
ACA for RVO	8	4.59 [2.75, 7.66]	0.00	0.720
ACA for CRVO	5	5.75 [2.25, 14.69]	0.00	0.854
ACA for BRVO	4	7.50 [1.61, 35.03]	27.80	0.245
LA for RVO	5	3.90 [0.99, 15.37]	37.00	0.890
LA for CRVO	4	1.71 [0.69, 4.21]	0.00	0.586

### Sensitivity analysis and publication bias

Each study was excluded and its effect on the main summary estimate and *χ* ^*2*^ test P value for heterogeneity was evaluated to assess whether a single study had a dominant effect on the meta-analytic OR. No single study significantly affected the summary estimate in this meta-analysis.

**Fig 4:**
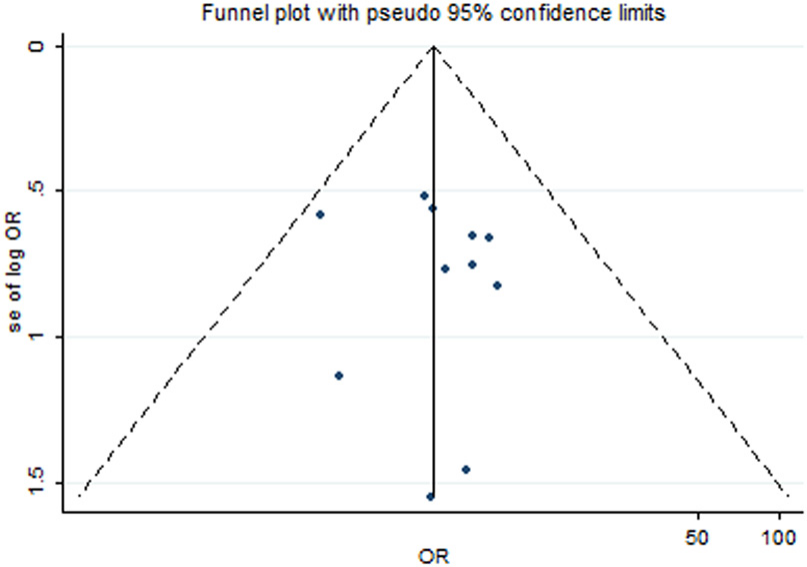
Funnel plot for assessment of publication bias.

The publication bias was assessed using visual inspection of funnel plots and Begg and Egger tests. No significant publication bias was found in the 11 selected studies (Begg’s funnel plot, symmetrical; Begg’s test, P for bias = 0.876; Begg’s test, P for bias = 0.717). The funnel plot is presented in Fig 4.

## Discussion

This systemic review and meta-analysis summarized the current literature and contained 11 case-control studies that investigated the association between APLA and risk of RVO. The pooled estimate from the random-effects model provides remarkable evidence of a significant association between APLA and RVO risk. Advanced subgroup meta-analysis showed that APLA was associated with increased risks of both CRVO and BRVO. However, APLA subtype analyses showed that it is ACA rather than LA was a risk factor of RVO.

APLA, including ACA and LA, are biomarkers of antiphospholipid antibody syndrome (APS). The association between APS and RVO has been studied in previous researches. For example, Ali et al. published a case report of BRVO and primary APS [27]. Therefore, it is important for us to detect the possible relationship between APLA expression and risk of RVO. APLA was also reported as an important biomarker for thrombophilia, thrombophilia and vascular risk factors. APLA was potentially important in the incidence of RVO [28,29]. A previous meta-analysis reported that congenital thrombophilic diseases were associated with the risk of RVO [28]. The possible relationship between APLA and thrombophilia might explain the increased risk of RVO in patients with positive APLA. More advanced studies are required to detect the association between vascular risk factors and the incidence of RVO. The results of this study would provide a better understanding of the risk factors of RVO.

Abuel-Asrar conducted a case-control study with 17 cases and 60 controls, and the results demonstrated that APLA was a risk factor of RVO in the younger group (less than 45 years old) [18]. These researchers also found a trend for the presence of APLA in CRVO patients with a poor visual acuity at presentation. These results suggest that the presence of APLA is a possible important factor for the development of RVO. Several studies were also conducted to detect the effects of ACA and LA independently. Glueck et al found that it was ACA but not LA was associated with an increased risk of RVO [20]. However, different results were detected in independent studies. Van et al. found that neither ACA nor LA was associated with the risk of RVO [16]. Meta-analysis is a useful statistical tool and the relevant studies are pooled together to gain a more powerful conclusion. A meta-analysis was also conducted to search for the potential risk factor of RVO. Kolar conducted a meta-analysis for the detection of the risks factor of RVO [28], and one basic risk factor for RVO was advancing age. An advanced study found that other risk factors, including systemic conditions such as hypertension, arteriosclerosis, diabetes mellitus, hyperlipidemia, vascular cerebral stroke, blood hyperviscosity, and thrombophilia, might be associated with an increased risk of RVO. This meta-analysis found that APLA was a risk factor of RVO, and advanced analyses showed that only ACA affected the incidence of RVO. However, this result was only marginally significant (OR = 3.90, 95% CI = [0.99, 15.37]), and only five studies were included in the analyses of LA and RVO risk. Therefore, this conclusion should be considered with caution. In this study, that ACA but not LA might be implicated in retinal vein occlusion. In the existing literatures, there are few studies on the relationship between LA and risk of RVO. Besides, the lower concentration and difficulty on the detection of LA might be the causes of the non-significant association between LA and risk of RVO. In the future, more studies are required on the association between LA and incidence of RVO. The advanced detection methods are also required.

This study reviewed the association between APLA and the risk of RVO. The main strength of this study is the detailed search strategy. The literature search included the MeSH and key word “risk factor”. It would be helpful to gain the possible inclusive data that are as complete as possible. Further advanced subgroup analyses would provide consummate knowledge on the issues of interests. Finally, the quantitative synthesis of data was considered clinically and statistically appropriate. This result would provide improved knowledge for clinical workers.

However, there are several limitations of this study. First, all of the included studies were case-control designs. The lack of blinding of sampling and data analyses might produce several potential biases. Second, no publication bias was detected using three independent methods, but a potential existing publication bias should also be considered. Accordingly, more well-designed and well-conducted epidemiological studies are required to deepen our understanding on the relationship between APLA and RVO incidence.

In conclusion, APLA, including ACA and LA, was significantly associated with the risk of RVO. The advanced analyses showed that ACA rather than LA affected on the risk of RVO. This study showed that APLA is a possible target for the detection of diagnostic and therapeutic interventions for RVO. Accordingly, additional well-designed and well-conducted epidemiological studies are required to deepen our understanding on the relationship between APLA and RVO risk.
