# Correction: Antiphospholipid Antibody and Risk of Retinal Vein Occlusion: A Systematic Review and Meta-Analysis

**DOI:** 10.1371/journal.pone.0157536

**Published:** 2016-06-09

**Authors:** Wei Zhu, Yan Wu, Ming Xu, Jin-Yu Wang, Yi-Fang Meng, Zheng Gu, Jiong Lu

There is an error in affiliation 2 for author Yan Wu. Affiliation 2 should be: Department of Ophthalmology, First Affiliated Hospital of Soochow University, Suzhou, China.
